# The Framework for Simulation of Bioinspired Security Mechanisms against Network Infrastructure Attacks

**DOI:** 10.1155/2014/172583

**Published:** 2014-08-31

**Authors:** Andrey Shorov, Igor Kotenko

**Affiliations:** ^1^Department of Computer Science and Engineering, Saint-Petersburg Electrotechnical University “LETI”, Professora Popova Street 5, Saint-Petersburg, Russia; ^2^Laboratory of Computer Security Problems, St. Petersburg Institute for Informatics and Automation, 14th Line 39, Saint-Petersburg, Russia; ^3^St. Petersburg National Research University of Information Technologies, Mechanics and Optics, Kronverkskiy Prospekt 49, Saint-Petersburg, Russia

## Abstract

The paper outlines a bioinspired approach named “network nervous system" and methods of simulation of infrastructure attacks and protection mechanisms based on this approach. The protection mechanisms based on this approach consist of distributed prosedures of information collection and processing, which coordinate the activities of the main devices of a computer network, identify attacks, and determine nessesary countermeasures. Attacks and protection mechanisms are specified as structural models using a set-theoretic approach. An environment for simulation of protection mechanisms based on the biological metaphor is considered; the experiments demonstrating the effectiveness of the protection mechanisms are described.

## 1. Introduction

Computer networks have become more targeted—large companies, public institutions, and critical infrastructures such as dams and nuclear stations can be under attack. Besides they are often notified about network worms and bots that generate large volume of traffic when spreading, overloading communication channels. According to reports of different companies, working in the field of computer security, the number of distributed attacks has increased significantly. For example the attacks such as “distributed denial of service” (DDoS) exceeded the capacity of 100 Gbit/s, while several years ago a typical DDoS attacks had the capacity about 10 Gbit/s. In 2013, the maximum power of these attacks for the first time exceeded 300 Gbit/s [1]. Thus, the necessity of the intensive research in network protection against infrastructure attacks (DDoS, worms, DNS attacks, and attacks against routers, etc.) is clearly seen.

Constant escalation of the power of attacks on the computer network infrastructure makes it increasingly difficult to protect against them through traditional security tools and generates a large number of errors in attack identification and determination of effective protection methods. Therefore, researchers are trying to develop new protection methods based on intelligent ways to detect and prevent attacks. Such solutions can be realized by security mechanisms based on the biological metaphor. Biological systems use a variety of security mechanisms in order to survive. By implementing and interpreting security mechanisms invented by nature, it is possible to bring in the area of computer networks effective ways of interaction, responses to the risks, and self-recovery in case of damage.

When designing and implementing new security systems based on bioinspired approaches, it is necessary to have efficient tools for their development, testing, analysis, and adaptation. The research of infrastructure attacks and protection mechanisms against them is rather complicated and robust process. To implement infrastructure attacks, large quantity of hosts united in one network is required. Infrastructure attacks are very dangerous; as in case of their implementation, the computer network can fail, and therefore it is impossible to observe such important conditions of scientific experiment as controllability and repeatability. Thus, simulation methods for analysis of infrastructure attacks and protection mechanisms seem to be the most preferable solution. The simulation provides a flexible mechanism for modeling complex dynamic systems that allows experimenting with different sets of parameters and scenarios, spending much less efforts, than in real networks.

The paper is an extended version of paper [2] and is devoted to the further development of the previously proposed bioinspired approach “network nervous system,” as well as methods of simulation of infrastructure attacks and protection mechanisms, which are based on “network nervous system.”

The main contribution of the work performed is the specification of formal model of the infrastructure attacks and protection mechanisms as well as the protection system based on biological metaphor which is called “network nervous system.” The implementation of simulation testbed is also presented. Experiments performed on simulation of infrastructure attacks and protection mechanisms are discussed, including the network nervous system protection mechanism.

The rest of this paper is organized as follows.  Section 2 considers related bioinspired approaches to protect from infrastructure attacks and methods of their simulation.  Section 3 specifies main models and algorithms to implement attacks and protection mechanisms based on “network nervous system.” Section 4 describes the implemented simulation testbed.  Section 5 outlines the experiments fulfilled and their evaluation. Finally, Section 6 presents main conclusions and future work directions.

## 2. Related Works

Research on development and application of approaches based on biological metaphors is quite extensive. Let us consider several papers relating bioinspired approaches that can be used for protection of computer networks.

In [3], three areas of possible usage of biological approaches in computer systems are highlighted: routing, security, and self-organization. In accordance with the scope of biological approaches the variants of the approaches that can be applied are selected.

“Ant” optimization algorithm is an approach that is used to optimize network traffic routing. The algorithm named AntNet [4] is now perhaps the most famous implementation of routing based on “ant” optimization algorithms.

A number of researchers is trying to reuse a set of approaches used in epidemiology. In [5], epidemiological algorithms are implemented to update a distributed database. In [6], methods for epidemic routing are proposed.

Hofmeyr and Forrest [7] suggest an approach based on the concept of immunnocomputing and the negative selection algorithm. The component that implements this approach works in analogy with antibodies that destroy all foreign objects in the human body. Dressler [8] as a basis for the mechanism of computer network protection uses an analogy with living cells. Anagnostakis et al. [9] propose a cooperative, based on immunology mechanism of protection against viruses named COVERAGE (cooperative virus response algorithm).

The focus of our paper is the further development of the approach called “network nervous system” which was proposed by Y. Chen and H. Chen. The protection mechanisms based on this approach consist of distributed procedures of information collection and processing, which coordinate the activities of the main devices of a computer network, identify attacks, and determine necessary countermeasures [10].

In order to conduct experiments on infrastructure attacks and protection mechanisms, we need tools allowing us to perform simulation of large-scale infrastructure attacks safely and with high adequacy. There are multiple works where simulation of various infrastructure attacks and protection mechanisms are used as the main research tool. These works are predominantly based on discrete event modeling of processes in network structures [11] and also trace-driven models in real computer networks [12].

In [13], experiments on worm propagation are conducted by Wagner et al. with the use of their own simulation system. In [14], Riley et al. employed the GTNetS simulation system for developing a network worm model.

In order to simulate propagation of the Slammer network worm [15] Suvatne created a model on the basis of the Wormulator simulation system [16]. Schuchard et al. [17] considered a simulation system development and simulation of network worm propagation using a model that consists of 250,000 nodes.

Protection mechanisms against such infrastructure attacks as network worm propagation are investigated. Consider the techniques based on virus throttling (VT) [18] and failed connection (FC) [19]. VT is based on limiting the number of new connections from a unique IP address to other IP addresses for the given time period. The FC technique involves TCP-connection analysis from a unique IP address. Packets with flags TCP RST and TCP SYN are monitored. If a host interrupts connection for a fixed period of time more often than a predetermined value, new connection requests from this IP address are limited.

Researchers pay much attention to protection mechanisms against attacks “distributed denial of service” (DDoS). Gamer and Mayer [20] perform a distributed mechanism of DDoS attack detection called Distack using the OMNeT++ simulation system. Distack is based on the ReaSE library intended for constructing realistic topology models, which correspond to a network of realistic patterns of legitimate network application traffic.

Li et al. [21] used their own simulation system and also testbeds for estimating efficiency, scalability, and cost of implementing the protection mechanism SAVE (Source Address Validity Enforcement) against DDoS attacks.

The present paper discusses the “network nervous system” metaphor as a bioinspired approach, which implements the main properties of a nervous system structure determining the mechanisms of information exchange, detection, and attack reaction. Special attention is paid to describing models and algorithms of their performance for the developed protection mechanism “network nervous system” with the help of a set-theoretic approach and pseudocode.

## 3. Models and Algorithms of Infrastructure Attacks and Protection Mechanisms

In this section, the main developed models that are used for implementation of “network nervous system” approach are specified. The structure of the models is described using a set-theoretic approach and algorithms of their work—using pseudocode.

### 3.1. Models of Base Mechanisms of Infrastructure Attacks

Set of models of infrastructure attacks is presented as follows:

(1) $$\begin{array}{c} M_{A}=\langle Sc,{En}_{A},{Ev}_{A},T\rangle , \end{array}$$

where *Sc* is a planner of simulation system, *En*_*A*_ are entities, related to infrastructure attacks models, *Ev*_*A*_ are events, generated by infrastructure attacks, and *T* is general modeling time.

Let us consider the following main elements of infrastructure attacks models:

(2) $$\begin{array}{c} {En}_{A}=\langle TA,CA\rangle , \end{array}$$

where *TA* is a type of infrastructure attack and *CA* is attack mechanism parameters.

Type of infrastructure attack may be given as

(3) $$\begin{array}{c} TA=\langle Wrm_{n,m},DDoS_{n,m},AoR_{n,m},AoDNS_{n,m}\rangle , \end{array}$$

where *Wrm*_*n*,*m*_ is network worms propagation, *DDoS*_*n*,*m*_ is distributed attack of “denial of service” type, *AoR*_*n*,*m*_ are attacks against routers, *AoDNS*_*n*,*m*_ are attacks against DNS servers, variable *n* serves for identification of attack type on the higher level of model representation, and *m* defines parameters for the given attack mechanism.

As an example let us consider the networks worms model. In order to create the network worms simulation model, we determine its representation as

(4) $$\begin{array}{c} Wrm_{n,m}=\langle I,M_{\text{ct}},f,Sprt,Dprt,M_{\text{st}},P_{\text{sc}},\text{PS},M_{\text{spoof}}\rangle , \end{array}$$

where *I* is worm identifier; for denoting network worms, the individual identifier is used; by using it we may uniquely determine a set of individual parameters of network worms; *M*_ct_ is connection type; connections may be of 2 types—based on TCP protocol or based on UDP protocol; *f* is frequency of packets generation, number of packets (connections) generated per second; *Sprt* is network port, from which packets are sent; *Dprt* is network port, to which packets are sent; *M*_st_ is scanning method; *P*_sc_ is probability of establishing successful TCP connection; the given parameter has its value in case of propagation of network worms according to TCP protocol; in case of work by UDP protocol, this parameter is marked as unavailable; PS is size of packet sent in the network; *M*_spoof_ is method of substitution of network address and port.

Events that may be generated by network worms are as follows:

(5) $$\begin{array}{c} Ev_{Wrm}=\langle nSpr,Vun\rangle , \end{array}$$

where *nSpr* is function responsible for beginning of worm propagation and *Vun* is function that determines work of the vulnerable node.

Let us consider the work of *nSpr* function. See Algorithm 1.

On timer triggering, a new timer is created that calls the function of packets generation with frequency *f*; then if connection type is TCP, attempt of connection with remote node is performed, where node is selected according to some scanning method *M*_st_. If connection is successful a packet that is responsible for remote node infection is sent. In case when connection type is not TCP, a packet performing vulnerability exploitation is being sent immediately.

Functioning of vulnerable host *Vun* is described as in Algorithm 2.

On receiving a packet, its useful load is extracted and the test, if it corresponds to the type of worm that can exploit vulnerability present in the given application, is performed. If the vulnerability is exploited, the node transfers to the state “infected” and calls the function *nSpr*.

### 3.2. Models of Base Mechanisms of Protection against Infrastructure Attacks

Set of base protection mechanisms which are used by the “network nervous system” approach is presented as

(6) $$\begin{array}{c} M_{D}=\langle Sc,En_{D},Ev_{D},T\rangle , \end{array}$$

where *Sc* is a planner of simulation system, *En*_*D*_ are entities of protection mechanisms, *Ev*_*D*_ are events by which protection mechanisms may operate, and *T* is modeling time.

Entities of protection mechanisms are as follows:

(7) $$\begin{array}{c} En_{D}=\langle TD,CD\rangle , \end{array}$$

where *TD* is the type of base protection mechanism, *CD* are parameters of protection mechanism.

Type of protection mechanism is as follows:

(8) $$\begin{array}{c} TD=\langle DWrm_{n,m},DfDDoS_{n,m},DAoR_{n,m},DAoDNS_{n,m}\rangle , \end{array}$$

where *D* *Wrm*_*n*,*m*_ are protection mechanisms against network worms propagation, *D* *f* *DDoS*_*n*,*m*_ are protection mechanisms against distributed attacks of “denial of service” type, *D* *AoR*_*n*,*m*_ are protection mechanisms against attacks on routers, *D* *AoDNS*_*n*,*m*_ are protection mechanisms against attacks on DNS servers, variable *n* serves for identification of protection mechanism on higher level of model representation, and *m* determines parameters of the given protection mechanism (e.g., location of the protection mechanism).

Protection mechanisms against network worms propagation are as follows:

(9) $$\begin{array}{c} DWrm=\langle TW,CW\rangle , \end{array}$$

where *TW* is type of the base protection mechanism and *CW* are parameters of the protection mechanism. As the base protection mechanisms can serve the mechanisms such failed connection, virus throttling, and so on. Then *D* *Wrm*_*n*,*m*_ = {*FC*, *VT*,…}, where *FC* is protection mechanism based on failed connection (FC) [19] and *VT* is protection mechanism based on virus throttling (VT) [18].

The protection mechanism based on the FC can be represented as

(10) $$\begin{array}{c} DWrm_{FC,m}=\left\{FC,\left(srcA,dstA,thr,bfP\left(adr,cP\right),bfBl\right)\right\}, \end{array}$$

where *srcA* is packet sender address; *dstA* is packet receiver address; *thr* is maximal number of received packets with flag RST set; *bfP*(*adr*, *cP*) is buffer of number of received packets with flag RST set for IP addresses of senders; *bfBl* is table of blocked addresses.

Let us define events of the FC protection mechanism as

(11) $$\begin{array}{c} Ev_{FC}=\left\{inPkt_{FC},Tm_{FC}\right\}, \end{array}$$

where event *inPkt*_*FC*_ calls function *PF*_*FC*_, which is executed at receiving packet from network; *Tm*_*FC*_ calls function *TF*_*FC*_, that describes actions of the protection mechanism at timer triggering.

The algorithm of function *PF*_*FC*_ can be represented as in Algorithm 3.

On packet receiving, the protection mechanism tests if the sender's address exists in the list of blocked addresses and in case of positive response it deletes the packet. If the sender's address is not in the list of blocked addresses, extraction of the TCP segment from the IP packet is performed. If control bits RST and ACK are set to “true” and “false,” respectively, search for the sender's address in the buffer of IP addresses of senders is done and if it is found, the number of available RST addresses is being decreased by 1. If available value is equal to or less than 0 the sender's IP address is added to the list of blocked ones. If IP address is not found in the buffer of sender's IP addresses, the IP address of packet source is recorded to this buffer.

Let us represent the work of *TF*_*FC*_ function. See Algorithm 4.

In a fixed period of time it is allowed to increase the number of permissible packets with flag RST by 1 (one) for all IP addresses in the buffer. This operation clears the list of blocked addresses since the number of permissible RST packets exceeds zero.

### 3.3. Protection Models Are Based on “Network Nervous System”

**Figure 1:**
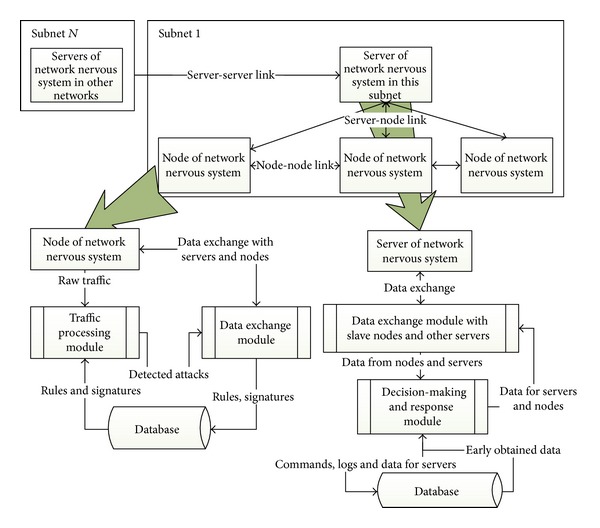
Common structural representation of the “network nervous system.”

The architecture of the system based on “network nervous system” can be represented as follows [2]. The domains of the network which are connected to “network nervous system” form an overlay network and communicate between each other via the VPN channels established between specialized security servers. The routers, located in different parts of the network, interact not only with each other, but also with their domain specialized security server (Figure 1).

The functionality of this architecture can be described at two levels: (1) local processing of obtained information by separate devices and (2) information processing within distributed cooperation of providers. The appointed security process is implemented locally on each node. Large-scale cooperation is fulfilled to implement protected information exchange in the domain (from a router to a router, from a router to the server), as well as between domains (from the server to the server). In this case information is automatically distributed between different nodes of the network.

Timely obtained information allows to respond on different external threats more effectively. Each node consists of functional blocks with the standard interface for data exchange. This provides flexibility when updating and maintaining nodes dynamically.

Now we will describe the main components of the “network nervous system” and algorithms of their performance. The “network nervous system” contains entities *En* and events *Ev* and also event scheduler and time axis. The entities are the “network nervous system” server and “network nervous system” node.

The “network nervous system” server contains a data exchange block with “network nervous system” nodes and data exchange block with other servers of the “network nervous system”, decision-making and response block and database.

Consider in a more detailed way the decision-making and response block. It has the following modules: prioritization module for received data; module of correlation between received data; module of data exchange with other servers of the “network nervous system” and local “network nervous system” nodes; module of information exchange with a server database; decision-making module.

The decision-making block can use the following functions: function characterizing the prioritization module performance; function characterizing the correlation module performance; function characterizing the blocking module performance; function for processing a local tree of an attack source; function for processing a global tree of an attack source.

The prioritization function works according to Algorithm 5.

The function Receive makes it possible for the component to receive *data*; if *data* contains suspicious IP addresses (*data* = IPLNod ∨ IPRServ), they have the highest processing priority *Prt*; *data* on suspicious packet passage (*data* = TLNod ∨ TRServ) has a lower priority; *data* from unknown sources is rejected.

After prioritization, the processing of received *data* is performed using the correlation function. See Algorithm 6.

If the module is free, it searches for data with the highest priority and performs search for information about addresses in a server database. These addresses are presumably those from which legitimate nodes are attacked. If a suspicious node has already been detected during such actions, threat level for this address increases. If there are no tasks with the highest priority, attack source identification is performed.

In order to determine attack sources the change aggregation trees (CAT) algorithm is used [22].

After attack source identification, the *bkTr* function is executed. See Algorithm 7.

This module checks whether or not the suspicious IP address has exceeded the threshold value *thr*. If the value has been exceeded, a decision is made to block the address of an attacker. This decision is transmitted to all subordinate nodes and also remote “network nervous system” servers.

Consider the components of the “network nervous system” node: module of information acquisition from sensors; module of data exchange with the “network nervous system” server; module of data exchange between “network nervous system” nodes; module of traffic processing implementation.

Now we will focus our attention on the traffic processing module, which consists of such components as flow-redirection block performing traffic division into flows according to a sender's and receiver's address; packet classification block determines a protocol and packet type (connection request and packet with data, etc.), analysis, and counteraction block.

Analysis and counteraction block can be represented as follows:

(12) $$\begin{array}{c} VD=\langle RlA,AnA,StA,TrBl\rangle , \end{array}$$

where *RlA* is the traffic analysis module on the basis of rules; *AnA* is the analysis module with the help of anomaly display module; *StA* is the signature analysis module; *TrBl* is the traffic blocking module.

Events generated by the analysis and counteraction block are as follows:

(13) $$\begin{array}{c} Ev_{VD}=\langle F_{NAnl},F_{\text{blck}},F_{tRA}\rangle , \end{array}$$

where *F*_*NAnl*_ is the function implementing traffic analysis; *F*_blck_ is the traffic blocking function; *F*_*tRA*_ is the function of malicious packet transmission to attack deterrence module.

Traffic analysis and blocking are implemented by the following way. See Algorithm 8.

*Rl* *A*′, *AnA*′, and *StA*′ are the rules, signatures, and commands used by the blocks *RlA*, *AnA*, and *StA*; *nDB* is a database node; *p* ^raw^ is a packet from a network, *p* ^mal^ is a malicious packet.

The traffic blocking function can be represented by the following way. See Algorithm 9.

## 4. Simulation Testbed

To assess the efficiency of the “network nervous system” a packet-level simulation testbed was created. It is based on the simulation framework described in [23, 24]. The simulation testbed architecture has four main components and includes the basic components of the “nervous network system,” the simulation models of infrastructure attacks and protection mechanisms in the form of application models.

The lower level is a basic simulation module. It is a simulation system based on discrete events. To simulate the Internet components and protocols, the Internet simulation module is used. It contains the components to generate the network topologies, the models of network applications, and protocols.

It uses also the library ReaSE, designed to simulate computer networks realistically representing the Internet. The subsystem of basic components of the “network nervous system” is a library that contains the components of the “network nervous system” and the general scenarios of the behavior implemented in the form of service and application models which are embedded in the models of computer network nodes. The module of domain processes includes the components of attacks and defense mechanisms, as well as the modules that implement the node functionality, including the filtering table, packet analyzer, and models of legitimate users. In the module of domain processes we specified the models of network worm propagation (including vulnerable node) and DDoS attacks.

The system includes the following functional subsystems:

- events simulation subsystem;
- integrated development environment;
- computer networks models generator;
- models of legitimate users;
- models of mechanisms of infrastructure attacks;
- models of mechanisms for protection against infrastructure attacks;
- models of interaction of mechanisms of attacks, protection, and legitimate users;
- subsystem for collecting output data from modules execution, subsystem for data analysis.

**Figure 2:**
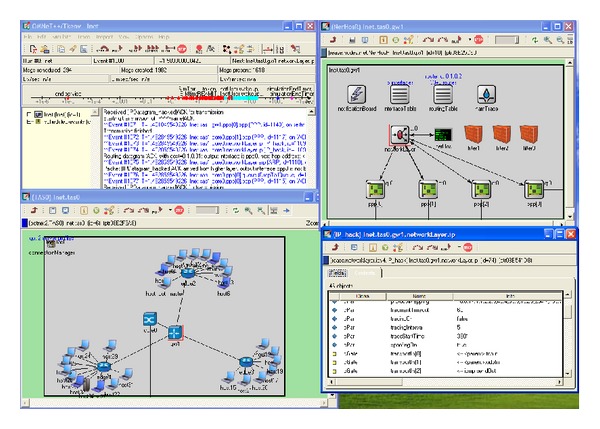
The interface of the simulation system.

Figure 2 shows the interface of the simulation system during experiments. In upper corner you can see the main panel that shows components that are included into general model and control elements allowing the user to interact with them. In the main panel we also have buttons allowing to control the simulation time; they allow, for example, to fulfill the model step by step or in accelerated mode. In addition, the control elements are presented that allow to perform efficient search of the needed object and subsequent change of its state. In the lower left corner a fragment of the simulated network is outlined.

Models of routers are denoted by cylinders with arrows; black rectangle is the server of “network nervous system”; hosts models are represented as computers of different colors. Colors are used to visualize host's state. Hosts that can be infected in case of network worms propagation are marked with blue; nodes that perform distributed attack of “denial of service” type are red. Legitimate nodes have no color identification. In the right upper corner there is the window of representation of the “network nervous system” node; under it we see the window showing its parameters and links.

The software tool developed provides researcher with functions which contain all necessary elements to simulate the systems for protection of computer networks from infrastructure attacks based on biological metaphor.

We chose the techniques based on FC [19], VT [18], SIM (source IP address monitoring) [25], and SAVE [21] approaches as basic protection mechanisms against infrastructure attacks and specified in the module of domain process the simulation models of these mechanisms as well as the protection mechanism based on “network nervous system.”

When experimenting only with the basic protection mechanisms, they are installed on all routers. When using the “network nervous system” approach, the basic protection mechanisms were connected to servers of the “network nervous system.” In both cases parameters of the computer network and the attack mechanisms were the same. This allowed us to compare the efficiency of the “nervous network system” with the basic protection mechanisms.

When the protection mechanisms operating independently, they apply own algorithms for attack detection and blocking. When they are included in the “network nervous system”, they not only implement their own detection mechanisms, but also send information about attacks to the corresponding server of the “network nervous system” and wait for the server response (commands) if no appropriate rule, signature or policy found.

## 5. Experiments and Evaluation

To carry out experiments, the network consisting of 3652 nodes was used. Ten of these nodes were servers. Among server nodes there were three web servers, one DNS server, and six mail servers. 1119 nodes (this is almost 30% of all nodes) had vulnerabilities that were necessary for successful network worm penetration; the same nodes were used to realize DDoS attacks.

The implemented experiments could be divided into two stages—efficiency assessment of the proposed protection mechanism against network worms (the first stage) and distributed DoS attacks (the second stage). The demonstrated results were partially presented at the PDP 2013 [2] and SIMULTECH 2012 [26] conferences.

For simulation of network worm propagation we made an assumption that some network hosts have vulnerabilities that can be exploited by the network worm. We assumed that the network worm uses the TCP protocol to spread and random scanning of IP addresses in given range to locate target hosts. To counteract the worm propagation, the protection mechanisms FC and VT were chosen as basic. When the “network nervous system” mechanism is switched on, these mechanisms are coordinated by the responsible server of the “nervous network system.”

**Figure 3:**
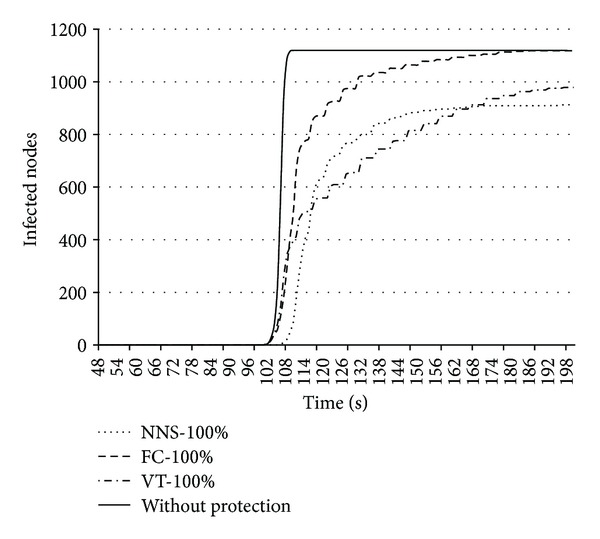
Number of the infected hosts depending on protection mechanism used (FC-100%, VT-100%, NSS-100%, or without protection).

Figure 3 demonstrates the number of infected hosts without any protection and the number of infected hosts depending on the chosen protection mechanism: FC mechanism installed on all routers (FC-100%), VT mechanism installed on all routers (VT-100%), and FC protection mechanism managed by “nervous network system” (NNS-100%).

It is clearly seen that in case of usage of FC mechanism managed by “network nervous system” the quantity of the infected hosts decreases compared to the FC and VT mechanisms.

To simulate DDoS attacks, we chose SYN flooding attack. In the half of the experiments we implemented sender IP address spoofing. To protect the network the mechanisms based on SAVE and SIM were used. The same protection mechanisms were used in conjunction with the “network nervous system.” Let us discuss some protection mechanisms.

The SAVE mechanism is set on a core router of a local subnet and allows identifying packets with spoofed address. When receiving a packet from an internal network, the SAVE checks whether the sender's address fits the range of the local network addresses. If it does not, the packet is rejected. Thus, the propagation of malicious packets to external networks is blocked.

**Figure 4:**
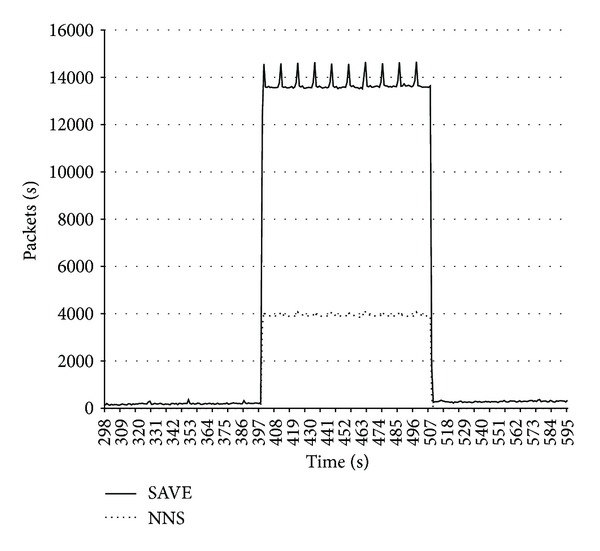
Volume of traffic on the attacked node in case of DDoS attack relative to simulation time.

Figure 4 shows amount of traffic on the attacked node when DDoS attack without the forging of the sender IP address is executed relative to the simulation time.

In the first case SAVE protection mechanisms are installed to protect from the DDoS attack. SAVE cannot detect malicious threads because attack is executed without the forging of the IP address.

In the second case SAVE and SIM protection mechanisms are connected to the “network nervous system.” In the protection mode SIM sends host addresses which are not present in trusted addresses database to the corresponding server of “network nervous system.” The local server distributes data about attacking nodes to other servers of the “network nervous system” which in their turn send the SAVE modules the command to block packets sent by attacking hosts.

The quality of the proposed “nervous network system” approach is estimated using the basic quality metrics such as false positive rate (FP), false negative rate (FN), true positive rate (TP), true negative rate (TN), and the additional metrics such as recall, precision, accuracy, mistake, and F-measure.

*Recall*. *r* is defined as the ratio of correctly classified packets to the total number of malicious packets: *r* = TP/(TP + FN).

*Precision*. *p* is calculated as the ratio correctly classified malicious packets to number of the all packets classified as malicious: *p* = TP/(TP + FP).

*Accuracy*. *a* is defined as the ratio of correct decisions made by system to the total number of decisions made by system: *a* = (TP + TN)/(TP + FP + FN + TN).

*Error*. *e* is calculated as the ratio of wrong decisions to total number of decisions made by system: *e* = (FP + FN)/(TP + FP + FN + TN).

*F-measure. F*_*i*_ is often used as general metric that unites recall and precision metrics: *F*_*i*_ = 2*p*_*i*_ *r*_*i*_/(*p*_*i*_ + *r*_*i*_).

We use also security metrics that characterize the overall efficiency of protection mechanisms. When evaluating the protection mechanisms against network worms the number of infected hosts (*N*_inf⁡_) is used. In case of DDoS attacks the volume of the malicious traffic coming on the attacked host (*V*_mal·traffic_) is assessed. The metrics that characterize the operation of basic protection mechanisms and the “network nervous system” mechanism (NNS) against network worm propagation are presented in Table 1.

Note that in this case “network nervous system” uses FC mechanism as the attack detector.

Table 1 shows that the protection mechanism FC managed by the “network nervous system” demonstrates better performance results in comparison with others when considering F-measure and number of the infected hosts.

The metrics that characterize the operation of basic protection mechanisms and the “network nervous system” mechanism against DDoS attacks are shown in Table 2.

Table 2 shows that the protection mechanism “network nervous system” also shows its worth in the case of protection against DDoS attacks with and witout substitution of a sender's IP address.

## 6. Conclusions

In the paper we offered to use the bioinspired protection mechanism “network nervous system” which is intended to counteract against infrastructure attacks. We proposed one of possible system architectures that implement such protection mechanisms and described main models and algorithms to implement attacks and protection mechanisms based on “network nervous system.”

To estimate the efficiency of the basic and proposed protection mechanisms, we developed the simulation testbed and carried out a set of packet level simulations. The implemented experiments showed the effectiveness of the basic security mechanisms managed by the “network nervous system” against such infrastructure attacks as network worm propagation and DDoS.

The presented paper shows the possibility of application different biological metaphors for network protection against infrastructure attacks. Certainly, not all biological approaches can be implemented today completely. However, many of them may be viable and give a new impetus to development of perspective security systems. Our next research steps will be devoted to improvement of the suggested approach and its comprehensive experimental evaluation.

## Figures and Tables

**Algorithm 1:**
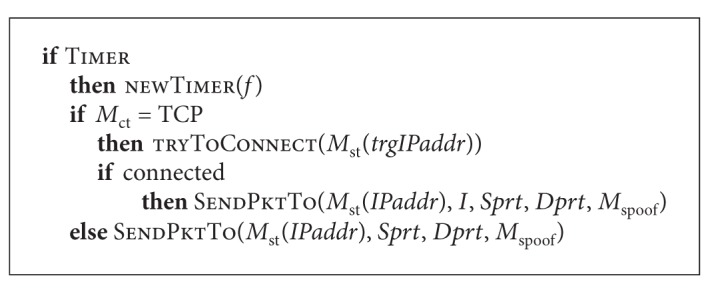


**Algorithm 2:**
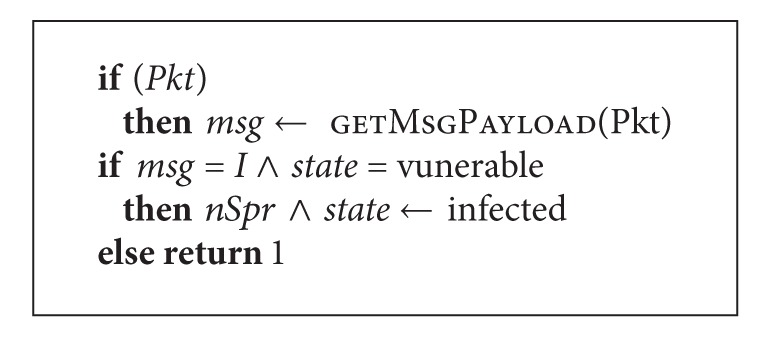


**Algorithm 3:**
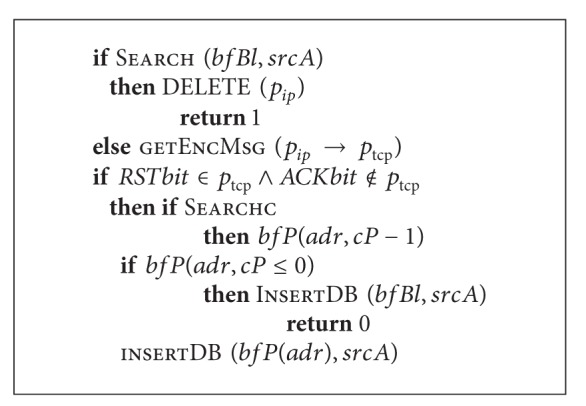


**Algorithm 4:**
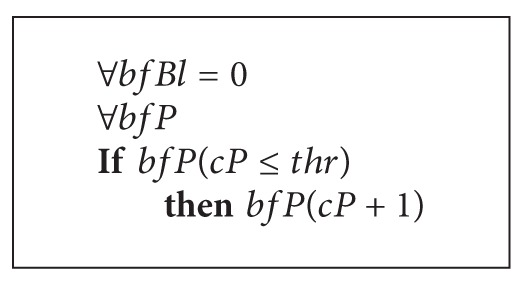


**Algorithm 5:**
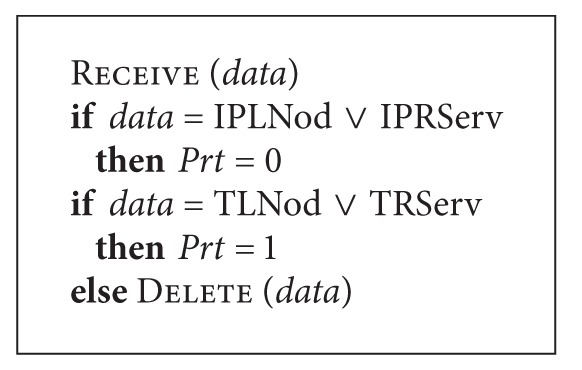


**Algorithm 6:**
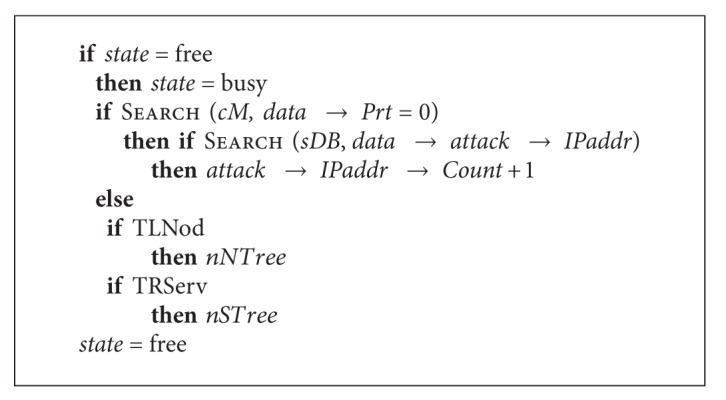


**Algorithm 7:**
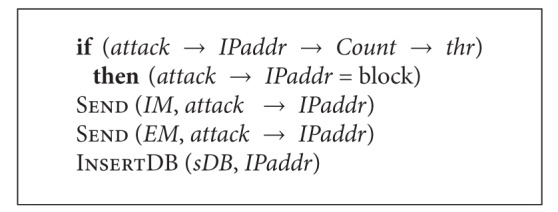


**Algorithm 8:**
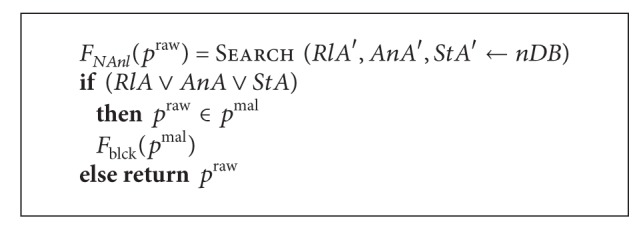


**Algorithm 9:**
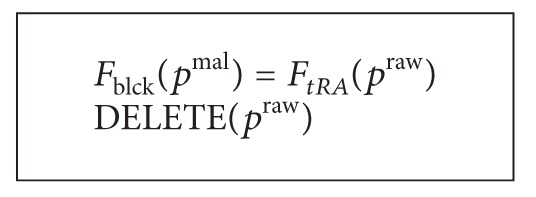


**Table 1:** Comparison of the protection mechanisms FC, VT, and NNS.

	FP	FN	*r *	*p *	*a *	*e *	*F*-measure	*N*_inf⁡_, %
FC	0.31	0.18	0.52	0.78	0.21	0.21	0.59	99
VT	0.01	0.57	0.43	0.98	0.66	0.33	0.60	93
NSS	**0.22**	**0.22**	**0.77**	**0.90**	**0.71**	**0.28**	**0.83**	**81**

**Table 2:** Comparison of the protection mechanisms SAVE, SIM, and NNS.

	FP	FN	*r *	*p *	*a *	*e *	*F*-measure	*V*_mal.traffic._, %
DDoS attack with sender IP address spoofing								
SAVE	0.04	0.01	0.99	0.97	0.98	0.02	0.98	1
SIM	0.09	0.01	0.99	0.98	0.97	0.03	0.98	99
NNS	**0.04**	**0.01**	**0.99**	**0.97**	**0.98**	**0.02**	**0.98**	**1**

DDoS attack without sender IP address spoofing								
SAVE	0.03	0.99	0.01	0.01	0.01	0.99	0.01	99
SIM	0.07	0.29	0.70	0.93	0.68	0.32	0.80	99
NNS	**0.07**	**0.30**	**0.69**	**0.93**	**0.68**	**0.33**	**0.80**	**30**
